# Design and Experimental Study of Space Continuous Robots Applied to Space Non-Cooperative Target Capture

**DOI:** 10.3390/mi12050536

**Published:** 2021-05-09

**Authors:** Yuwang Liu, Dongqi Wang, Yongchao Zhang, Zhongqiu Yuan, Jinguo Liu, Sheng Yang, Yi Yu

**Affiliations:** 1 State Key Laboratory of Robotics, Shenyang Institute of Automation, Chinese Academy of Sciences, Shenyang 110016, China; wangdongqi@sia.cn (D.W.); zhangyongchao@sia.cn (Y.Z.); yuanzhongqiu@sia.cn (Z.Y.); liujinguo@sia.cn (J.L.); yangsheng@sia.cn (S.Y.); yuyi@sia.cn (Y.Y.); 2 Institutes for Robotics and Intelligent Manufacturing, Chinese Academy of Sciences, Shenyang 110169, China

**Keywords:** continuous robot, space capture, scissor mechanism

## Abstract

Space capture actuators face problems such as insufficient flexibility and electrical components that are vulnerable to extreme space environments. To address these problems, a centralized-driven flexible continuous robot based on a multiple scissor mechanism units is proposed in this study. The continuous robot body is composed of two scissor mechanism units coupled in series, and the base container’s three motors to drive the robot. The two scissor mechanism units ensure a wide range of flexible operations and the light weight of the robot. The centralized drive with three motors not only reduces the number of driving sources, but also ensures temperature control and protection of electrical components in the space environment. The kinematics and dynamics of the robot are analyzed, and the workspace and deformation performance of the robot are verified through experiments. Compared with other continuous robots, the proposed continuous robot retains the characteristics of continuous robots in a wide range of flexible operations. At the same time, the configuration is light and a small number of driving sources are used, which is suitable for extreme temperatures, vacuum, radiation, and strict resource-constrained environments in space.

## 1. Introduction

**Figure 1:**
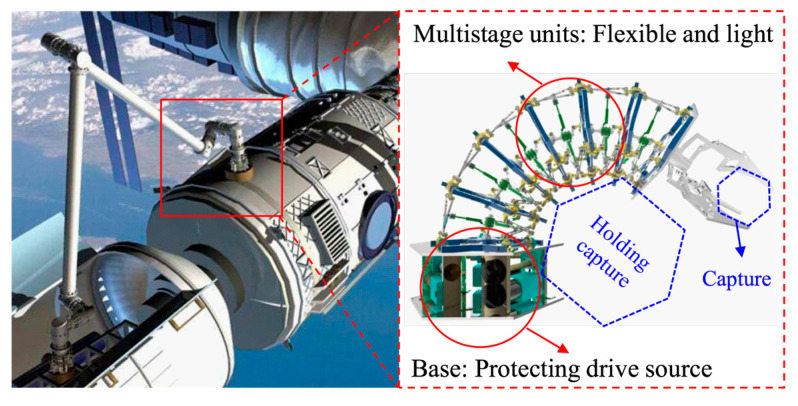
Schematic diagram of space continuous robot and capture mode.

With the development of global space science and technology, space has gradually become crowded. The safety of spacecrafts in orbit will be seriously affected by abandoned spacecrafts, various space debris, and other non-cooperative targets. In order to ensure the safety of spacecraft, non-cooperative target acquisition in space has been increasingly investigated by researchers. The space capture actuator is generally used as one of the tool sets, installed at the end of a large space robot arm. In extreme space environments, space capture actuators have the advantages of flexible bending, strong adaptability to non-structural environments, and resistance to extreme space environments. Continuous robots have obvious advantages in the field of non-cooperative target capture due to their high redundant degrees of freedom and flexibility. Continuous robots can use end grippers to capture objects like traditional industrial robots and can use the entire robot body to achieve holding capture as shown in Figure 1. 

Continuous robots can be subdivided into discrete redundant robots, soft robots, and rigid continuous robots. Compared with discrete redundant robots and soft robots [1,2], rigid continuous robots are more rigid and are expected to be used in space capture tasks. According to the driving mode, rigid continuous robots can be divided into three categories: rope drive, concentric-tube drive, and fluid drive.

Rope-driven continuous robots are composed of driving ropes and multiple units, and bend as the rope stretched. Walker et al. established the theoretical framework of the bionic continuous robot, and developed a variety of continuous robots that imitate an elephant’s trunk [3,4]. OCRobotics Ltd. (Bristol, UK) developed a multi-free serpentine operating arm with strong obstacle avoidance ability and high load capacity [5]. Camarillo et al. developed a continuous manipulator based on rope drive, which has been used in medical fields such as cardiac catheters [6]. Lenny et al. built a multi-section robot with a follow-the-leader motion, with which the robot reached the bronchi wall of a human lung [7]. Xu et al. successfully developed a super-redundant flexible robot prototype that can operate in extreme environments such as narrow spaces and nuclear radiation environments [8,9].

Concentric-tube-driven continuous robots are composed of a special alloy, which realizes the bending of the robot by controlling the deformation of the alloy. Wang et al. designed a three-arm concentric tube robot, which was used in nasopharyngeal carcinoma surgery [10]. Rone designed a super-elastic rod continuous robot composed of eight discs [11]. Dwyer et al. proposed a concentric-tube continuous robot used for treating twin-twin transfusion syndrome [12].

Fluid-driven continuous robots use gas or liquid in closed cavities to maintain the shape of the robot and are usually lighter weight. Walker et al. developed Air-Octo [13] and OctArm [14] to realize bending and concertina movement. The German company Festo developed an elephant-trunk continuous robot driven by pneumatic pressure, which assists people in complex tasks [15]. Ranzani et al. designed a pneumatic-driven continuous robot, which realized bending and concertina motion by controlling three cavities [16]. Kang et al. proposed an air-filament hybrid mechanism to achieve the bending control of a flexible continuous robot [17,18]. Li et al. designed a pneumatic-driven variable stiffness continuous robot, which could flexibly grasp a variety of samples [19].

In summary, continuous robots have achieved many successful applications in specific environments due to their multiple degrees of freedom and flexibility. However, there are still some shortcomings when they are applied to the unstructured space environment [20]. First, continuous robots are driven by fluids or ropes, which are difficult to work in extreme temperatures, vacuums, and radiation environments for a long time. Second, the existing continuous robots have huge energy requirements, where each degree of freedom movement on average needs 1.5 driving sources. Finally, the control of a continuous robot is complicated, and the large amount of calculations poses a considerable challenge to the space chip [21].

To address the problem that existing continuous robots cannot be used in extreme environments in space, this study proposes a centralized-driven flexible continuous robot based on multiple scissor mechanism units. In this work, Section 2 introduces the design and structure of the proposed continuous robot. The kinematics of the continuous robot is analyzed in Section 3. In Section 4, the experiment platform of the continuous robot is built, and experiments are carried out to verify the workspace and deformability of the continuous robot. Section 5 discusses existing problems. The conclusion is provided in Section 6.

## 2. Design of Space Continuous Robot

The proposed space continuous robot is mainly composed of two parts, the base and multistage units. The multistage units are mainly composed of scissor mechanism units, which improve the flexibility of the system. At the same time, due to the application of the linkages, the continuous robot has lightweight characteristics. The base is mainly composed of drive motors and linear slide rails. The base effectively guarantees the precision of electrical parts such as motors, and controllers are protected from the harsh space environment.

### 2.1. Design of the Scissor Mechanism Unit

**Figure 2:**
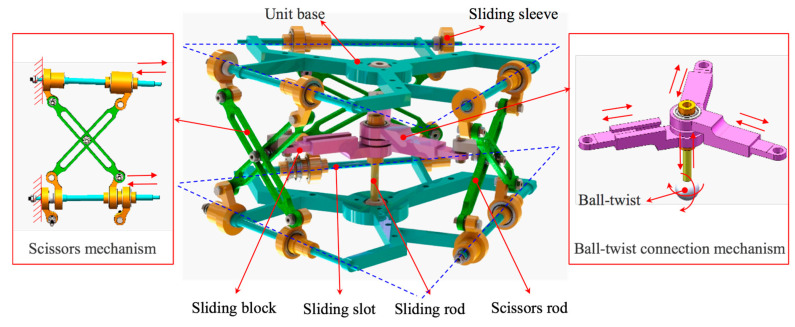
Model of scissor mechanism unit.

As shown in Figure 2, the main composition of the scissor mechanism unit is the scissor mechanism and the ball-twist connection mechanism. Each scissor mechanism unit has three scissor mechanisms, which are separately connected to the unit bases of the upper and lower scissor mechanism units, and are distributed in an equilateral triangle, as shown by the blue dashed line. One side of the scissors mechanism is fixed, and the other side is a sliding sleeve, which can slide horizontally along the unit base. In order to ensure the stability of the mechanism, a ball-twist connection mechanism was designed. The ball-twist connection mechanism connects to the center of the unit base by a ball-twist, which can realize spherical rotation. The sliding rod is connected to the ball-twist and rotates with the ball-twist. Three sets of sliding slots and sliding blocks are connected to the end of the sliding rod. One end of the sliding block can slide straight into the sliding slot. The other end of the sliding block is fixed at the hinge point of the scissors mechanism. When the scissors mechanism unit moves, three sliding sleeves move horizontally on the unit base, and three sets of scissor mechanisms are coupled to move, then the ball-twist connection mechanism rotates together with the scissor mechanisms. The scissors mechanism unit can realize the functions of stretching, compressing, and deflecting.

When multiple units are connected in series to form the space continuous robot and the corresponding sliding sleeves are moved horizontally on the unit base together, the movement transmission and flexible large-scale deformation are realized.

### 2.2. Design of the Base

**Figure 3:**
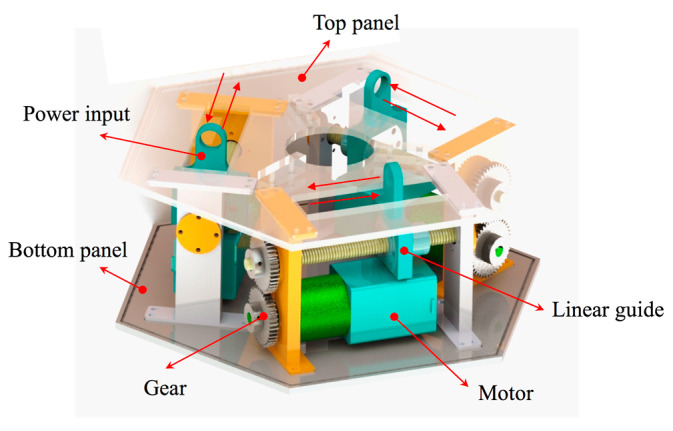
Model of the base.

The base is composed of three sets of motors, gears, and linear slides. The motor is connected to the gear to rotate, and the gear drives the linear slide to move. The slider on the linear slide is the power input, and the top panel is slotted to move the slider out. The power input is connected to the sliding sleeve on the scissor mechanism units, which drive the movement of the multistage units. Since all electrical parts are placed in the base, reasonable electromagnetic and temperature control protection of the base can ensure system stability in extreme space environments (Figure 3).

## 3. Kinematics Analysis

### 3.1. Workspace Analysis

**Figure 4:**
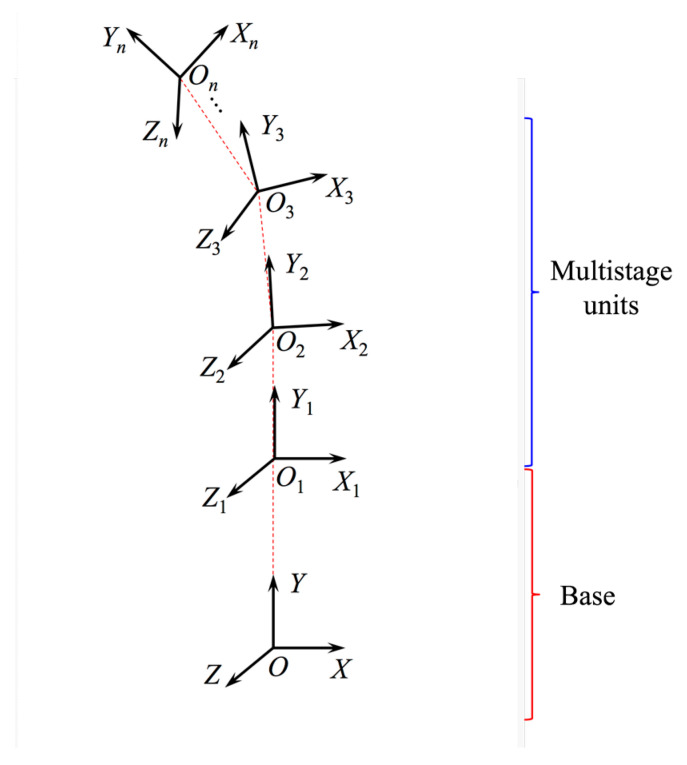
Establishment of the coordinate system of the continuous robot model.

In order to simplify the work space of the continuous robot, the robot was regarded as a link-type joint robot. The space continuous robot model is shown in Figure 4. A Cartesian system was established at the base origin [22], and Denavit-Hartenberg (D-H) parameters of the series multiple units are shown in Table 1. In order to verify the prototype system, only 2 units were calculated.

The motion transformation model of a single scissor mechanism unit was established.

(1) $${}_{B}^{A}T=\mathrm{Trans}{(}^{A}p_{B_{0}})\mathrm{Rot}(k,\theta )=\left[\begin{array}{cc} \begin{array}{l} I_{3\times 3}\\ 0 \end{array} & \begin{array}{l} {}^{A}p_{B_{0}}\\ 1 \end{array} \end{array}\right]\left[\begin{array}{cc} {}_{B}^{A}R\left(k,\theta \right) & 0\\ 0 & 1 \end{array}\right]$$

where ${}_{B}^{A}T$ is the homogeneous transformation matrix of one joint, which describes the position and posture of the coordinate system {***B***} relative to the coordinate system {***A***}; Trans (${}^{A}p_{B_{0}}$) is the translation transformation matrix, where the position vector ${}^{A}p_{B_{0}}$ describes the position of the origin of the coordinate system {***B***}; Rot (***k***, *θ*) is the rotation transformation matrix, which represents the rotation operator of the deflection angle *θ* of the axis ***k*** through the origin; the rotation matrix ${}_{B}^{A}R\left(k,\theta \right)$ describes the posture of the coordinate system {***B***} relative to the coordinate system {***A***}; and $I_{3\times 3}$ is a 3 × 3 identity matrix.

From the homogeneous transformation matrix, the transformation matrix of each joint was obtained as:

(2) $$\left\{ \begin{array}{l} {}_{1}^{0}T=\mathrm{Trans}\left(0,a_{0},0\right)\mathrm{Rot}\left(z,\theta_{0}\right)\\ {}_{2}^{1}T=\mathrm{Trans}\left(0,a_{1},0\right)\mathrm{Rot}\left(z,\theta_{1}\right)\\ {}_{3}^{2}T=\mathrm{Trans}\left(0,a_{2},0\right)\mathrm{Rot}\left(z,\theta_{2}\right)\\ {}_{4}^{3}T=\mathrm{Trans}\left(0,a_{3},0\right)\mathrm{Rot}\left(z,\theta_{3}\right) \end{array}\right.$$

Because each unit is connected in series to form the continuous robot, the deflection angle *θ* of each unit is the same, which means that *θ_i_*(*i* = 0, 1, 2, 3) = *θ*. According to the D-H method [22], a 4 × 4 homogeneous transformation matrix is used to describe the spatial relationship between two adjacent links, and each link transformation matrix can be obtained from Table 1.

(3) $${}_{1}^{0}T=\left[\begin{array}{cccc} 1 & 0 & 0 & 0\\ 0 & 1 & 0 & 0\\ 0 & 0 & 1 & 0\\ 0 & 0 & 0 & 1 \end{array}\right]\left[\begin{array}{cccc} c\theta & -s\theta & 0 & 0\\ s\theta & c\theta & 0 & 0\\ 0 & 0 & 1 & 0\\ 0 & 0 & 0 & 1 \end{array}\right]$$

(4) $${}_{2}^{1}T=\left[\begin{array}{cccc} 1 & 0 & 0 & 0\\ 0 & 1 & 0 & a\\ 0 & 0 & 1 & 0\\ 0 & 0 & 0 & 1 \end{array}\right]\left[\begin{array}{cccc} c\theta & -s\theta & 0 & 0\\ s\theta & c\theta & 0 & 0\\ 0 & 0 & 1 & 0\\ 0 & 0 & 0 & 1 \end{array}\right]$$

(5) $${}_{3}^{2}T=\left[\begin{array}{cccc} 1 & 0 & 0 & 0\\ 0 & 1 & 0 & a\\ 0 & 0 & 1 & 0\\ 0 & 0 & 0 & 1 \end{array}\right]\left[\begin{array}{cccc} c\theta & -s\theta & 0 & 0\\ s\theta & c\theta & 0 & 0\\ 0 & 0 & 1 & 0\\ 0 & 0 & 0 & 1 \end{array}\right]$$

(6) $${}_{4}^{3}T=\left[\begin{array}{cccc} 1 & 0 & 0 & 0\\ 0 & 1 & 0 & a\\ 0 & 0 & 1 & 0\\ 0 & 0 & 0 & 1 \end{array}\right]\left[\begin{array}{cccc} c\theta & -s\theta & 0 & 0\\ s\theta & c\theta & 0 & 0\\ 0 & 0 & 1 & 0\\ 0 & 0 & 0 & 1 \end{array}\right]$$

The pose relationship between the robot end and the base was obtained from the pose matrix of the robot end in the coordinate system of the base.

(7) $${}_{4}^{0}T={}_{1}^{0}T{}_{2}^{1}T{}_{3}^{2}T{}_{4}^{3}T=\left[\begin{array}{cccc} r_{11} & r_{12} & r_{13} & r_{14}\\ r_{21} & r_{22} & r_{23} & r_{24}\\ r_{31} & r_{32} & r_{33} & r_{34}\\ 0 & 0 & 0 & 1 \end{array}\right]$$

where $r_{31},r_{32},r_{13},r_{23},r_{33},r_{34}=0$ and

(8) $$r_{11}=c\theta \left(c\theta \left(c{\left(\theta \right)}^{2}-s{\left(\theta \right)}^{2}\right)-2c\theta s{\left(\theta \right)}^{2}\right)-s\theta \left(s\theta \left(c{\left(\theta \right)}^{2}-s{\left(\theta \right)}^{2}\right)+2c{\left(\theta \right)}^{2}s(\theta )\right)$$

(9) $$r_{21}=c\theta \left(s\theta \left(c{\left(\theta \right)}^{2}-s{\left(\theta \right)}^{2}\right)+2c{\left(\theta \right)}^{2}s\theta \right)+s\theta \left(c\theta \left(c{\left(\theta \right)}^{2}-s{\left(\theta \right)}^{2}\right)-2c\theta s{\left(\theta \right)}^{2}\right)$$

(10) $$r_{12}=-c\theta \left(s\theta \left(c{\left(\theta \right)}^{2}-s{\left(\theta \right)}^{2}\right)+2c{\left(\theta \right)}^{2}s(\theta )\right)-s\theta \left(c\theta \left(c{\left(\theta \right)}^{2}-s{\left(\theta \right)}^{2}\right)-2c\theta s{\left(\theta \right)}^{2}\right)$$

(11) $$r_{22}=c\theta \left(c\theta \left(c{\left(\theta \right)}^{2}-s{\left(\theta \right)}^{2}\right)-2c\theta s{\left(\theta \right)}^{2}\right)-s\theta \left(s\theta \left(c{\left(\theta \right)}^{2}-s{\left(\theta \right)}^{2}\right)+2c{\left(\theta \right)}^{2}s\theta \right)$$

(12) $$r_{14}=-as\theta -a\left(s\theta \left(c{\left(\theta \right)}^{2}-s{\left(\theta \right)}^{2}\right)+2s\theta c{\left(\theta \right)}^{2}\right)-2ac\theta s\theta ,$$

(13) $$r_{24}=ac\theta +a\left(c\theta \left(c{\left(\theta \right)}^{2}-s{\left(\theta \right)}^{2}\right)-2c\theta s{\left(\theta \right)}^{2}\right)+a\left(c{\left(\theta \right)}^{2}-s{\left(\theta \right)}^{2}\right).$$

The workspace of the space continuous robot was calculated approximately by forward kinematics. On the basis of the analysis of forward kinematics, Monte Carlo method was used to analyze the robot workspace.

The position vector of the robot end position was obtained in the base coordinate system by forward kinematics. Then, the Rand () function in MATLAB was used to generate a series of random numbers (0,1), and we used random numbers to form random *p_x_* and *p_y_*.

**Figure 5:**
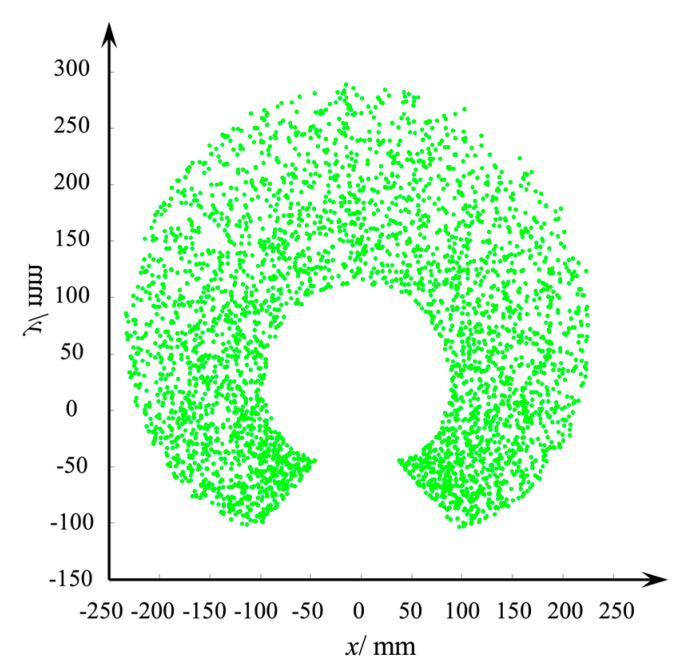
Workspace of the space continuous robot.

The range of *θ* was assumed to be (20°,−20°), and 100,000 random points were selected. The calculated robot workspace is shown in the Figure 5.

The workspace of the continuous robot composed of two scissor mechanism units on the *XOY* plane is shown in Figure 5. Because the workspace solving method of the link-type joint robot was adopted, the influence of the base was not considered. The robot can reach from −250 to 250 mm in the *X* direction and theoretically can reach from −50 to 300 mm in the *Y* direction. In the actual movement process, the *Y* direction cannot reach below 0 mm. Due to the limitation of the robot structure, the circular space about 100 mm from the origin cannot be reached. If the number of scissor mechanism units is increased, the robot workspace will further enlarge.

### 3.2. Motion Control Parameter Analysis

**Figure 6:**
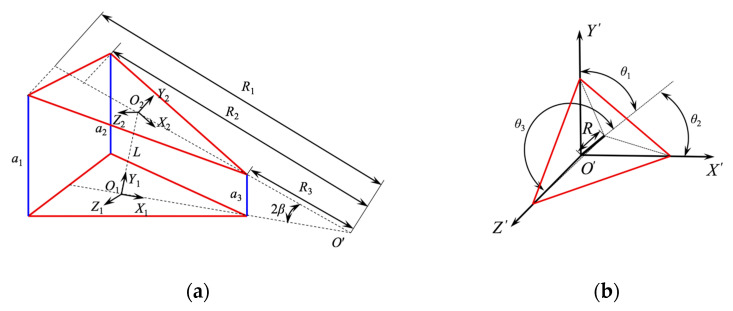
Simplified structure of the scissor mechanism unit. (**a**) Stretch structure simplification. (**b**) Deflection structure simplification.

To realize the motion control of the space continuous robot, the relationship between the input variable and the angle change needed to be accurately obtained. The robot is driven by three motors evenly distributed along the circumferential direction at 120°. The movement of the robot is realized by changing the state of the scissors mechanism through the input control. The simplified structure of the scissor mechanism unit is shown in Figure 6.

Regarding *a*_1_, *a*_2_, and *a*_3_ as the input of the three motors, the motors directly control the changes in *a*_1_, *a*_2_, and *a*_3_. *β* is the turning angle of the scissor mechanism unit to the origin *O*’. *θ*_1_, *θ*_2_, and *θ*_3_ are the deflection angles of the sliding rod to the axes of *Y*’, *X*’, and *Z*’, respectively. *L* is the distance between the center points of two scissor mechanism units. *R* represents the distance from the unit base to the sliding slot. *R*_1_, *R*_2_, and *R*_3_ represent the cosine value of the trilateral output to the origin *O*’. Δ*R* is the change in *R* with the input.

According to the geometric relationship in Figure 6, the following relationship was obtained,

(14) $$L=\frac{\left(a_{1}+a_{2}\right)}{12}+\frac{a_{3}}{3}$$

(15) $$R=\frac{L}{2\sin\beta }$$

(16) $$R_{i}=R-\Delta R_{i}=R-\Delta R\cos\theta_{i}(i=1,2,3)$$

(17) $$a_{i}=2R_{i}\sin\beta$$

Bringing Equations (15)–(17) into Equation (14),

(18) $$L=a_{1}+2\Delta R\cos\theta_{1}\sin\beta =a_{2}+2\Delta R\cos\theta_{2}\sin\beta =a_{3}+2\Delta R\cos\theta_{3}\sin\beta$$

According to the structure of the scissor mechanism unit, the relationship between the deflection angle *θ* and *θ*_1_, *θ*_2_, and *θ*_3_ is as follows,

(19) $$\theta_{1}=-\theta ,\theta_{2}=\frac{2\pi }{3}-\theta ,\theta_{3}=\frac{4\pi }{3}-\theta$$

then,

(20) $$L=a_{1}+2\Delta R\cos\theta \sin\beta =a_{2}+2\Delta R\sin\beta \left(-\frac{1}{2}\cos\theta +\frac{\sqrt{3}}{2}\sin\theta \right)$$

(21) $$L=a_{1}+2\Delta R\cos\theta \sin\beta =a_{3}+2\Delta R\sin\beta \left(-\frac{1}{2}\cos\theta -\frac{\sqrt{3}}{2}\sin\theta \right)$$

The deflection angle *θ* can be obtained by combining Equations (20) and (21) as follows,

(22) $$\theta =arc\tan\frac{\sqrt{3}(a_{2}-a_{3})}{2a_{1}-a_{2}-a_{3}}$$

Therefore, the deflection angle *θ* can be determined according to the input of *a*_1_, *a*_2_, and *a*_3_.

## 4. Experiments

**Figure 7:**
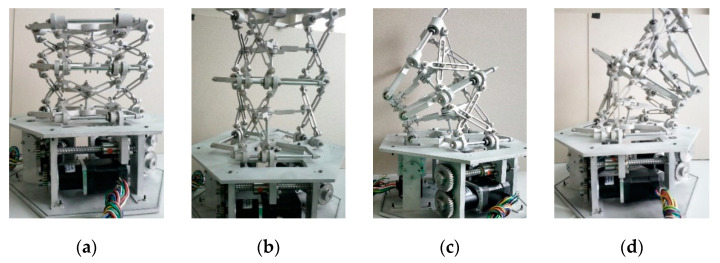
Prototype of the space continuous robot: (**a**) initial contraction state, (**b**) stretch state, (**c**) ultimate bending state (left), and (**d**) ultimate bending state (right).

The design parameters are shown in Table 2, with the ultimate deflection angle of 20°. The space continuous robot prototype is shown in Figure 7, and the parts were all composed of aluminum alloy, in which Figure 7a is the initial contraction state, Figure 7b is the stretch state, and Figure 7c,d is the ultimate bending state.

**Figure 8:**
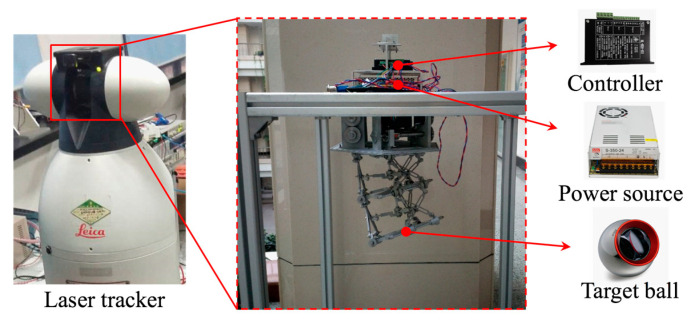
Experimental platform.

In order to verify the robot control performance, the experimental platform shown in Figure 8 was designed. The prototype was reversely clamped on the aluminum frame to reduce the influence of structural gravity on the end position. The NL-WSH-V01 controller, power of S-350-24, and a No. 45 stepper motor and its components were used to stick the target ball at the center of the scissor mechanism unit end. The Leica laser tracker (Leica Geosystems AG-Part of Hexagon, St. Gallen, Switzerland) was used to track the position of the target ball, which obtained the space coordinates of the center point, and then converted it into the deflection angle *θ*.

**Figure 9:**
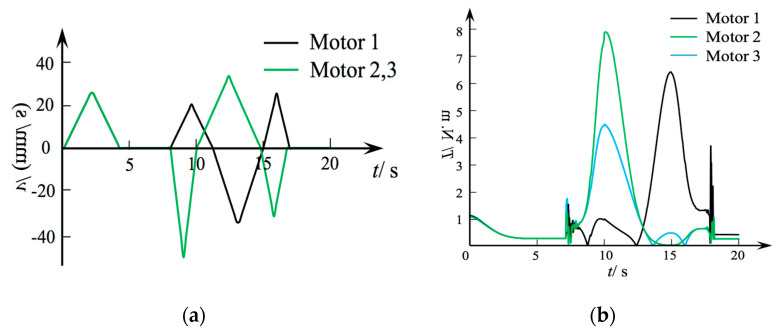
Input speed and torque of three motors. (**a**) Input speed vs. time curve. (**b**) Input torque vs. time curve.

The continuous robot turned to the limit value on one side, and then reversed to the limit value on the other side. The motor speed and torque changes with time were recorded as shown in Figure 9a,b. The forward movement of the three motors at the same time in the first 5 s extended the continuous robot the longest distance. From 8 to 10 s, motor 1 continued to rotate, and motors 2 and 3 rotated in the reverse direction, making the continuous robot move in the direction of the center of the motor 2 and 3 positions. From 10 to 15 s, motor 1 rotated in the reverse direction and motors 2 and 3 rotated in the forward direction, so that the continuous robot turned to the limit position of motor 1. From 15 to 17 s, motor 1 rotated forward, and motors 2 and 3 rotated in the reverse direction, which returned the continuous robot to the extended state.

Figure 9b shows the torque output of the three motors. From 0 to 5 s, the output torque of three motors was basically the same, indicating that the system was stable during the extending process. The robot bent and changed from the extended state at 7 s. At this time, the starting torque of the motor fluctuated to a certain extent. At 10 s, the torque error between the two motors was larger. Ideally, the torques of motor 2 and motor 3 should be exactly the same, but the prototype had assembly errors and was influenced by system friction, which affects the motor to a certain extent during the bending process. At 15 s, there was a certain error in the output torque of motors 2 and 3. Finally, at 17 s, the motor stopped working, but due to hysteresis characteristics and gap errors, there was still a certain torque value.

**Figure 10:**
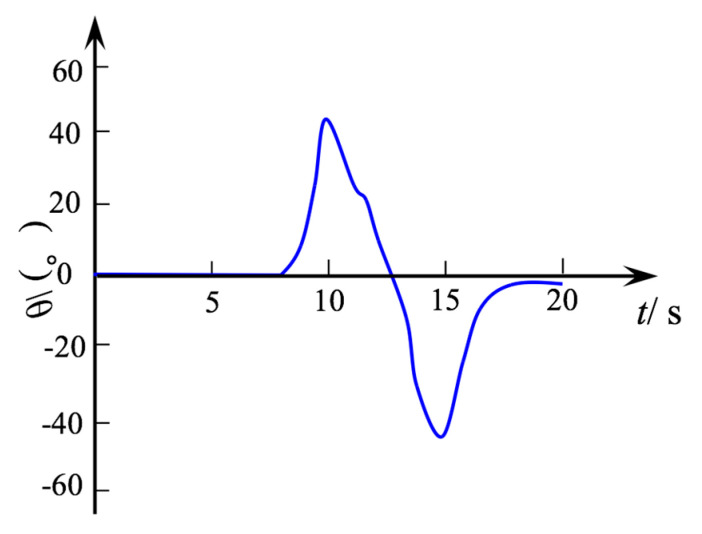
The measured deflection angle with time.

The target ball was tracked by the laser tracker to obtain the end position of the robot. The position was converted into the rotation angle of the robot and the fitting curve is shown in Figure 10. It can be seen that the maximum of the rotation angle as about ±40°, which is consistent with the design limit angle. From 7 to 10 s, when the robot moved to one side, the movement was smooth. From 10 to 15 s, in the process of moving to the other side, it can be seen that the rotation angle changed and there was a minor tremor, which occurred due to the effect of the gap in the assembly process. From 15 to 17 s, the robot returned back to the extended state; however, the deflection angle *θ* did not return to 0°, which also confirmed that the gap had a slight impact on the robot system.

## 5. Discussion

In this work, a novel space continuous robot with large range movement ability was constructed. Through the combination of theoretical analysis and experiment, the feasibility of the movement of two scissor mechanism units and its workspace was verified. Due to gravity on the ground, friction has a huge impact on the robot system. Especially when the multi-units are connected in series, the superposition of friction force increases the motor’s burden and the change in rotation angle trembles further. Therefore, the next step will be to consider expanding the number of scissor mechanism units from two to six or seven in a microgravity environment, which will further expand the workspace of the space continuous robot. At the same time, a claw will be designed at the end to verify the holding and capturing performance.

## 6. Conclusions

This research proposed a flexible continuous robot that can be applied to capture non-cooperative targets in space and has a large range of movement in all directions. With the shortcomings of existing continuous robots, which cannot be used in the space environment, a base containing all of the electrical components of the drive was designed, where temperature control can effectively protect the fragile components from extreme temperatures. The scissor mechanism unit, which combines the scissors mechanism and ball-twist connection mechanism, was used, and the continuous robot was constructed through the series connection of the multistage units and the base. The continuous robot can realize a wide range of three degrees of freedom flexible motion under the drive of three motors. Kinematics analysis of the continuous robot was carried out, and the workspace of the continuous robot was determined. The control parameters of the robot were analyzed, and the motion control variables of the continuous robot were derived. The prototype of the continuous robot and experiment platform was built and the experiments to analyze the relationship between motor speed and torque over time were carried out. The relationship between the deflection angle and time was measured by a laser tracker. The ability of the continuous robot to stretch, bend, and perform large-scale motion were verified, and the existing errors were discussed.

## Figures and Tables

**Table 1:** D-H parameters of space continuous robot.

Link.	*θ*	*α*	*a*_i−1_	*d*_i_	Range of *θ* (°)	Range of *a* (mm)
1	*θ*	0	0	0	(–20,20)	0
2	*θ*	0	a	0	(–20,20)	(32–80)
3	*θ*	0	a	0	(–20,20)	(32–80)
4	*θ*	0	a	0	(–20,20)	(32–80)

**Table 2:** Design parameters of scissor mechanism unit.

Design Parameters	Connecting Rod of Scissor Mechanism	Single Side of the Base	Stroke of Slide Sleeve	Stroke of Slide Block
Distance (mm)	80	93	100	19
